# Endometriosis node in Gynaecologic scars: A study of 17 patients and the diagnostic considerations in clinical experience in tertiary care center

**DOI:** 10.1186/s12905-015-0170-9

**Published:** 2015-02-18

**Authors:** Rocío Vellido-Cotelo, Jose L Muñoz-González, Maria R Oliver-Pérez, Cristina de la Hera-Lázaro, Cristina Almansa-González, Concepción Pérez-Sagaseta, Jesús S Jiménez-López

**Affiliations:** Endometriosis Unit, Universitary Hospital, 12 de Octubre, Madrid, Spain

**Keywords:** Cutaneous endometriosis, Incisional endometriosis, Scar endometriosis, Perineal endometriosis

## Abstract

**Background:**

Endometriosis nodes are observed in extra pelvic locations, particularly in gynaecological scars, with the abdominal wall being one of the most frequent locations. The main objective of the study is to review patient characteristics of cases of endometriosis nodes in gynaecological scars.

**Methods:**

A retrospective, observational and descriptive study with a cohort of patients from Hospital 12 de Octubre was conducted from January 2000 to January 2012. We analysed all of the patients who presented with an endometriosis node in a gynaecological scar presentation who had undergone surgery in that period. Descriptive data were collected and analysed.

**Results:**

A total of 17 patients with an anatomopathological diagnosis of an endometriosis node in a gynaecological scar were found. The following variables were studied: the age at diagnosis (32.5 years +/− 5.5 years), personal and obstetric history, time from surgery to diagnosis (4.2 years +/− 3.4 years), symptoms (a painful mass that grows during menstruation is the most frequent symptom in our patients), technical analyses by computed tomography (CT), magnetic resonance (MR) or fine needle aspiration (FNA) (77% of the patients), node size (2.5 cm +/− 1.1 cm) and location (caesarean scar, 82%; episiotomy scar, 11.7%; and laparoscopic surgery port, 5.8%), involvement of adjacent structures (29% of the patients), treatment (exeresis with a security margin in all the patients) and other endometriosis locations (14% of the patients).

**Conclusions:**

A high level of suspicion is required to diagnose gynaecological scar endometriosis, which should be suspected in the differential diagnosis of scar masses in reproductive-aged women.

Several theories have been proposed to explain the formation of endometriosis nodes in extrauterine localizations. The two of them that seem to be more plausible are the metaplasia and transport theories.

Imaging with ultrasound, CT and MR facilitate the diagnosis. FNA could be used for preoperative diagnosis.

Treatment must be by node resection with a security margin. In some cases, surgery could be combined with hormonal treatment.

**Electronic supplementary material:**

The online version of this article (doi:10.1186/s12905-015-0170-9) contains supplementary material, which is available to authorized users.

## Background

Endometriosis is defined as the finding of endometrial glands and stroma outside the uterine cavity. This tissue is found in ectopic pelvic organs, such as the ovaries, vaginal straight union, escrow bladder or bladder, as well as in extra pelvic locations as the lungs, kidneys, ureters or brain [1]. The abdominal wall is one of the most frequent extra pelvic locations [2] of endometriosis.

Endometriosic implants are found in the subcutaneous tissue of surgical scars, more frequently after procedures performed during pregnancy [3], including diagnostic amniocentesis. They are found in non-gynaecological surgery scars as those from appendectomies or umbilical hernioplasties [4]. Endometriosis in an episiotomy scar is more rare (occurring in only 0.00007% of births) [5,6]; however, recent studies indicate that the actual incidence is underestimated [7].

The theory of direct implantation is widely accepted by many authors [8,9]. Other theories argue that an endometriosic node in scars occurs because of scar tissue metaplasia (primitive mesenquimal cells would differentiate to endometrial cells), and other hypothesis defends migration through lymphatic or vascular vessels to distant sites. The metaplasia and migration theory could explain distant nodes in sites without direct contact with endometrial tissue [8-11].

The great variability in the clinical presentation and limited knowledge of the disease cause difficulty in diagnosing endometriosic nodes in scars, especially among specialists who do not normally treat this type of patient [12]. Imaging techniques such as CT, MR or ultrasound assist in identifying the condition; however, pathology examination of a node is required for diagnostic confirmation [13,14].

In our study, we analysed the cases of endometriosis in gynaecological procedure scars that occurred during a 12-year period in the Hospital 12 de Octubre and performed a review of the existing literature.

## Methods

We performed a descriptive, observational, retrospective study in a cohort of patients in Hospital 12 de Octubre in the 12-year period from January 2000 to January 2012 and aimed to analyse all the patients diagnosed with endometriosis in gynaecological scars who received surgery in that period. The study was approved by the local Ethics Committee of 12 Octubre Hospital. Madrid. Spain (110/2013). The analysed variables are described in Table 1, including the patient age, medical history, clinical features, location, time between surgery and the onset of the nodule, diagnostic method, location, involvement of other structures adjacent to the endometrioma, existence of endometriosis in other locations and treatment of the injury. All the patients underwent pathologic confirmation of the diagnosis. All patients signed informed consent authorizing the publication of their clinical data as well as the corresponding images.

**Table 1:** **Patients characteristics, diagnostic methods and treatment performed**

**Patients**	**Age at Diagnosis**	**Personal History**	**Time between Surgery and Diagnosis**	**Symptoms**	**Image Diagnosis/FNA**	**Location**	**Involving Neighboring Structures**	**Node Size**	**Other Location Endometriosis**
1	29	Laparoscopic ovarian cystectomy for endometriosis (1999). Ewing sarcoma in right psoas (2000) treated with chemotherapy and radiotherapy. Wedge resection of right ovary for endometriotic cyst (Same time surgical psoas)	10	Dark skin lesion that festers in umbilical region in previous scar area of 2x1cm. Dysmenorrhea.	MR/No	Infraumbilical	No involvement. Fascia was resected for cancer.	2 cm	Yes. Ovary.
		G0P0							
2	38	Without positive findings	3	Painful node in episiotomy. The pain is accompanied by inflammation and always coincides with menstruation. Pain increases as time passes.	Ultrasound/No	Deeply in episiotomy scar. It extends into the ischiorectal fossa.	No	3 cm	No
		G1V1 Forceps							
3	32	Without positive findings	2	Episiotomy painful node which size increases with menstruation.	No/Yes: endometriosis	Episiotomy.	No	1.5 cm	No
		G1 V1							
4	28	Endometriosis diagnosed in 2007.	4	Intense dysmenorrhea. Abdominal wall nodule in right rectus.	Ultrasound/Yes: endometriosis	Right rectus of the abdomen muscle.	Yes. Nodule in right hemiabdomen.	1 cm	Yes. Ovary.
		G1 C1: active genital Herpes virus lesions.							
5	36	Without positive findings	3	Painful node.	Ultrasound/No	Right angle cesarean scar.	No	1.5 cm	No
		G2 A1 C1: No progress in labor							
6	32	Without positive findings	3	Painful tumor of 4–5 cm in the upper left corner of cesarean scar.	No/Yes: endometriosis	Left angle of cesarean scar	No	5 cm	No
		G2 C1 V1 (2008): suspected fetal distress							
7	23	Without positive findings	2	Tender lump in cesarean section (right paramedian region) that increases in size with menstruation.	Ultrasound/No	Rigth angle of cesarean scar	Fascia and muscle involvement.	1 cm	No
		G1 C1: breech presentation.							
8	39	Resection of endometriotic node in cesarean section in 2005. Recurrence in 2010.	2	Tender lump in the upper left corner of cesarean section, which increased in size.	Ultrasound/Yes: no titratable.	Left angle of cesarean scar.	Fascia involvement.	1.5 cm	No
		G1 C1: breech presentation							
9	35	Without positive findings	5	Nodule in left corner of cesarean scar which hurts and comes bigger with menstruation	Ultrasound/Yes: endometriosis	Left angle of cesarean scar.	No	3 cm	No
		G1C1 cephalic-breech twin							
10	45	Without positive findings	15	Umbilical tumor that increases with Valsalva maneuvers.	No/No	Umbilicus. Medium laparatomy scar.	Hernia sac	2 cm	No
		G2 A1 C1 breech							
11	37	Hemithyroidectomy for multinodular goiter. Hypothyroidism. Appendectomy	5	Dysmenorrhea. Hypermenorrhoea.	No/No	Abdominal oblique muscle.	Yes	2 cm	No
		G4 A2 V1 C1:suspected fetal distress							
12	33	Tuberculosis in 1994. Anxious depressive syndrome. Migraines.	1	Tumor that appeared after an effort in hypogastrium, which is propelled by coughing and reduced manually. About infraumbilical laparotomy scar. Approx 4 cm	No/No	Down the umbilicus in infraumbilical laparotomy scar.	Hernia sac	3 cm	No
		G2 C2: breech presentation and uterine septum/anterior cesarean section and transverse situation in 2001.							
13	27	Without positive findings	1.5	Tumor that increases in size in left iliac fosse after cesarean section.	CT/Yes. Carcinoma. Intraoperative biopsy: endometriosis. Definitive result: endometriosis.	Left iliac fosse, related to cesarean section scar.	Resection is performed removing surrounding tissue until peritoneum.	3 cm	No
		G1C1: breech presentation							
14	29	Without positive findings	5	Nodule in right angle cesarean scar that hurts and comes bigger with menstruation	No/Yes: endometriosis	Right angle of cesarean scar.	No	3 cm	No
		G1 C1: No progress in labor							
15	30	Appendectomy	4	Tender lump in the upper left corner of cesarean scar	No/Yes: endometriosis	Left angle of cesarean scar.	No	2 cm	No
		G1 C1: transverse situation.							
16	36	Without positive findings	5	Bluish nodule in left corner of cesarean scar	No/Yes: endometriosis	Left angle of cesarean scar.	No	1 cm	No
		G2 V1 C1: No progress in labor							
17	26	Without positive findings	3	Node in left iliac fosse that increases in size with menstruation.	No/No	Left iliac fosse, related to cesarean section scar.	No	4 cm	No
		G2 A1 C1: breech presentation							

We conducted a comprehensive review of the existing literature on the subject.

## Results and discussion

We searched the databases of the Pathology Department and selected all the entries identified as endometriosis in the abdominal wall and perineum in the period from January 2000 to January 2012, confirming the pathologic diagnosis of endometriosis in 17 cases.

**Figure 1:**
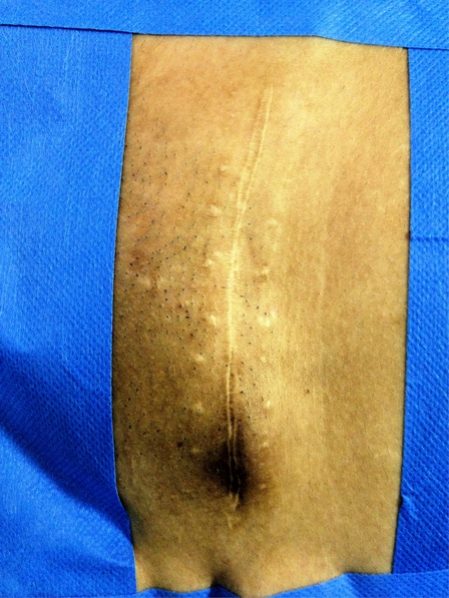
**Bluish nodule in left corner of cesarean scar.**

**Figure 2:**
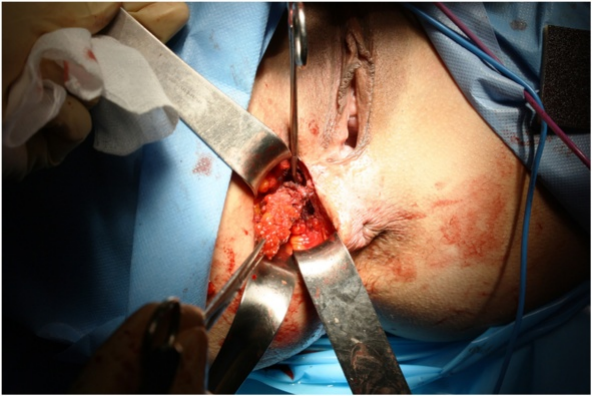
**Endometrioma in episiotomy.**

**Figure 3:**
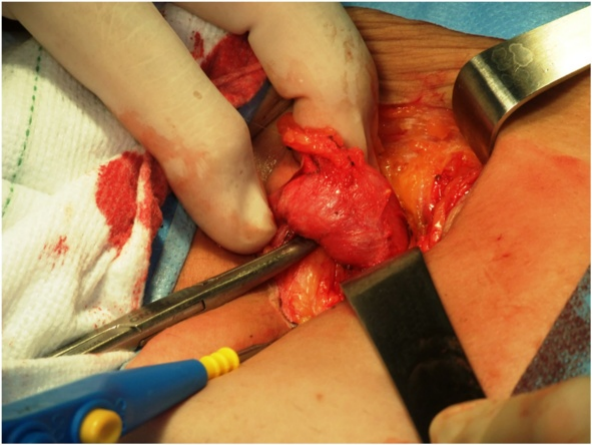
**Umbilical endometriosis in laparoscopic trocar acces scar.**

Of the 17 patients diagnosed with gynaecological scar endometriosis, a previous caesarean section had been performed in 14 women (82.3%) (Figure 1); in 2 patients, the antecedent procedure was an episiotomy (Figure 2) (11.7%), and 1 patient had a laparoscopic cystectomy of endometriotic ovarian cysts (5.8%). Within the group caesarean section cases, 2 patients each had an endometriotic nodule in an umbilical hernia observed in an infraumbilical laparotomy (Figure 3). Relapse occurred in one patient (5.8%), and surgery was performed.

The mean obstetric history of the patients was 1(+/− 0.54) gestation. The mean age of the patients at diagnosis was 32.5 years (+/- 5.5 years; Range: 23–45). The average time between the antecedent event and the appearance of the surgical endometriotic nodule was 4.2 years (+/- 3.4 years; Range: 1–15).

The clinical presentation varied with the location of the node. The main symptom of a caesarean scar nodule was the finding of a palpable mass (82%) that increases in size with menstruation (47%) and is painful (41%); 18% of the patients have dysmenorrhea. In two of the patients, the clinical finding was the appearance of an umbilical hernia scar involving an infraumbilical caesarean section.

In the 2 cases involving an episiotomy scar nodule, the clinical presentation was the appearance of a painful node in the perineum, which enlarges and causes pain with menstruation. Regarding the endometriotic nodule in an umbilical scar following trocar access, the clinical presentation was a node that festered with menstruation.

**Figure 4:**
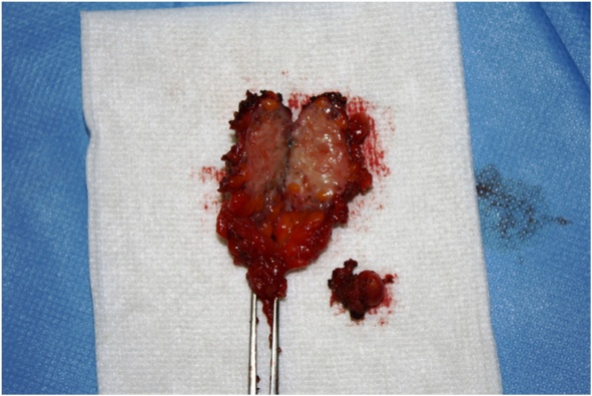
**Endometrioma: resected specimen.**

**Figure 5:**
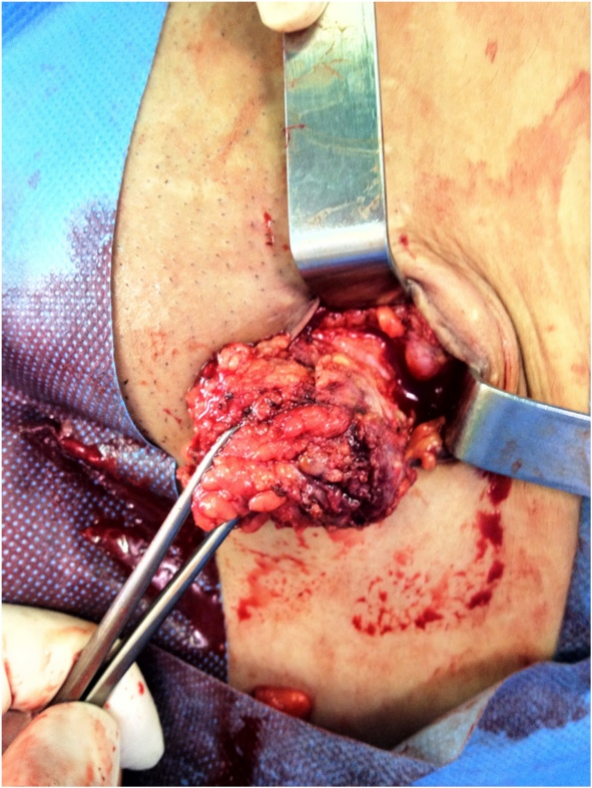
**Excision of left cesarean section angle endometriosic node with security margin.**

The average tumour size was 2.5 cm (+/- 1.1 cm; Range 1–5) (Figure 4). Regarding the node location, 47% of the patients had a nodule in direct relation to a scar, whereas 53% of the nodes were found essentially adjacent to and in the muscle plane lateral to the incision. In 5 of the 15 patients (29%) with an abdominal wall nodule, resection of the fascia plane adjacent to the lesion was required to allow an appropriate safety margin; in 4 of the patients, a mesh placement was specified to correct the defect (Figure 5). All the patients underwent resection of the tumour and subsequent pathological diagnostic confirmation.

**Figure 6:**
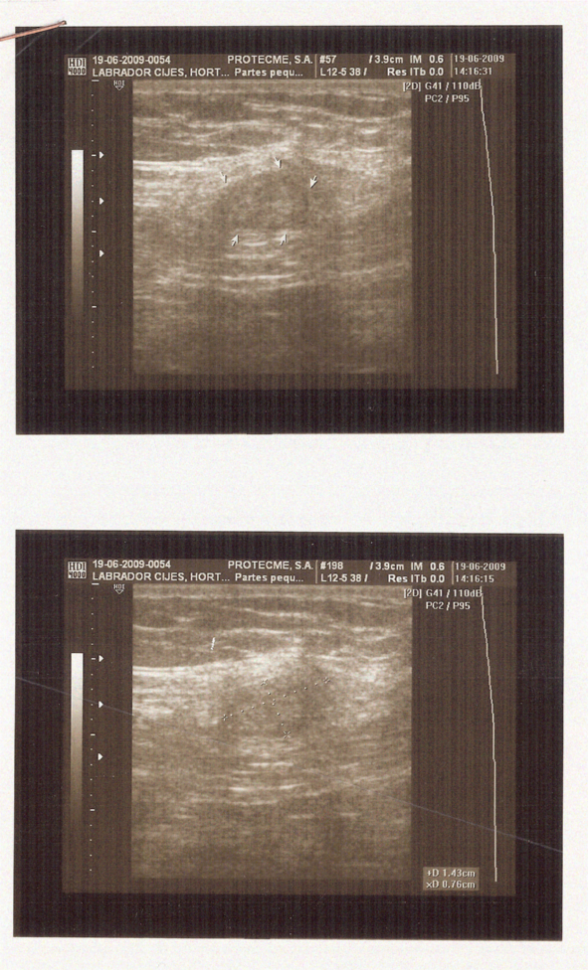
**Ecographic image of an endometriosic node.**

In 52% of the patients, a pathologic diagnosis was obtained before surgery by performing FNA. Additionally, 47% of the patients were provided a complementary imaging test for the diagnosis, including ultrasound in 75%, MR, in 12.5% and CT, in 12.5% of the patients (Figure 6).

In 23% of the cases, the diagnosis was made clinically. Two of the 17 patients (14%) had associated endometriosis in another location, which was ovarian endometriosis in both cases.

We observed that the pathological diagnosis was confirmed in 100% of the patients who underwent surgery for a suspected endometriosic scar node.

The differential diagnoses of the clinical presentations of this condition include diverse presentations such as granuloma, incisional hernia, haematoma, abscess, cyst or lipoma, causing the diagnosis of endometrioma to be difficult [4,15].

The diagnosis of endometriosis in a gynaecological scar is made clinically if other classical clinical signs are present [16]. The presence of a mass (96%) or pain (87%) is the most common symptom. Other common symptoms are bleeding in superficial lesions or hypogastric pain [1,4]. In our study, the most common symptoms were consistent with those described in the literature, with the appearance of a palpable mass (82%), pain (41%) and a size increase of the mass concurrent with menstruation (47%). We found literature references to dysmenorrhea as a symptom in these patients; however, in our study 21% of the patients presented with dysmenorrhea, and two of these patients had pelvic endometriosis.

Endometriotic nodules on gynaecological scars appear in patients of childbearing age (15–55 years), and a mean age of onset of 31 years was found in the literature, which is similar to that found in our study (a mean age of 32.5 years).

The time interval between surgery and the occurrence of the symptoms described in the literature is between 45 days and 20 years [16-18]. According to a review of 29 published articles by Horton et al. the mean time to the onset of symptoms is 3.6 years (95% CI 2.5-4.8) [1]. In our study, the average time was 4.2 years.

There was an association with pelvic endometriosis in 13% of the patients [1]. This percentage is similar to that found for pelvic endometriosis in patients of childbearing age (8-15%) [19], and it appears that the incidence of pelvic endometriosis in patients with endometriomas in gynaecological scars is similar to that in the general population. Our study is consistent with the literature, in that of our patients, 14% have associated pelvic endometriosis.

Our institution has no standardized protocol for preoperative diagnostic testing in cases of nodes in gynaecological scars. Imaging techniques such as ultrasound, magnetic resonance imaging or computed tomography facilitate the diagnosis. Ultrasonography is a good test because it is cost-effective [4,8,20]. The typical ultrasound finding is a solid, hypoechoic and vascularised nodule with speculated margins infiltrating the surrounding tissue. CT and MR facilitates a diagnosis in cases of large masses, by providing observation of the relationship of the mass to the neighbouring structures. Computed tomography is a poor imaging modality because of the lack of resolution and radiation exposure [21]. The imaging techniques do not provide a definitive diagnosis [16,20,22]. In our study, 47% of the patients provided the results of an imaging test, which was an ultrasound test in 75% of the cases.

The use of FNA is controversial. Some authors assert that this technique increases the risk of producing new endometriotic implants in the puncture site and the risk of viscera injury (a differential diagnosis of endometrioma is incisional hernia) [4,22].

However, others defend this technique arguing that is an accurate method to make the diagnosis before the surgery, so it is possible to avoid errors in the approach of the abdominal wall endometriosis scars and helps to plan the best treatment. The use of this technique provides a pathologic diagnosis before surgery in cases of diagnostic uncertainty regarding the origin of a mass [1]. Constant findings are described in these cytologies such as glandular epithelial cells, spindle or ovoid stromal cells and a hemorrhagic background with hemosiderin-laden macrophages [23-26].^.^Tru cut biopsy seems not to have a clear role in these cases because of the accuracy of FNA in most patients and the risks related to some differential diagnosis such as hernias. Discarding these considerations, it may be take into account to confirm malignancy in suspicious cases [27]. In our patients, 52% had a FNA diagnosis before surgery. One of our patients was diagnosed with cancer by this method, and subsequently the final pathologic examination not confirmed. These lesions, which are influenced by the hormonal cycle, increase in volume as non-invasive tissue, which correlates with histopathological changes that cause the endometriotic tissue to appear to be tumour-like [4].

In general, malignancies in endometriomas in gynaecological scars are rare, occurring in 0.31% of cases. The most common histological type is clear cell carcinoma, with a survival at 20 months of 57% [28]. Malignancy should be suspected in cases of frequent recurrence or in fast-growing large endometriomas [28,29].

Regarding the pathogenesis of the disease, there are several hypotheses: metaplasia, migration and direct contact, they have been explained before. Nowadays we still do not know yet which is the right one, but it is probably is a mixture of them.

It is described that gynaecological scar endometriomas appear much more frequently in caesarean sections, than episiotomies [30,31]. According to Wicherek et al., the appearance of a scar endometrioma after a caesarean section increases in caesarean sections performed without an active phase of labour or after the initiation of labour; this difference was statistically significant [32]. In our study, 64% of the caesarean sections were performed prior to the onset of labour, which supports the data in the study of Wicherek.

Many authors support the etiopathogenic hypothesis of iatrogenic implantation of endometrial cells during surgery [8,33,34]. This approach explains one case in our study, in which a nodule was found in the umbilical trocar incision scar from a laparoscopic bilateral ovarian cystectomy for endometriosis, with no access to the uterine cavity.

This theory alone does not explain the pathophysiology of this process, and the explanation requires a multifactorial etiological hypothesis.

Regarding the treatment, there is a consensus that there should be node resection that maintains safety margins [1,20], which is the most successful procedure, with the lowest probability of recurrence; the final diagnosis requires an accurate pathologic analysis [20,22]. A wide resection of a nodule requires inclusion of a segment of the adjacent structures such as the fascia or muscle. In these cases, most authors repair the wall by placement of a mesh [1,2,4], which occurred in 4 of the patients in our study.

The option of medical treatment alone does not appear to be effective. Most patients have a recurrence of symptoms after stopping treatment. There might be an option for a pre-surgical approach in cases in which the node is largely endometriotic or concurrent with pelvic endometriosis [35]. The drugs most commonly used are oral contraceptives, danazol, leuprolide and progesterone [1].

There is a question as to whether endometriomas in gynaecological scars are preventable. There are no consistent data in the literature to support any preventive measure. Hypotheses have been suggested such as externalising the uterus at the time of opening the cavity, extra abdominal suturing of uterine incisions, thorough cleaning of the abdominal cavity after the procedures setting the endometrium, closing the parietal peritoneum or changing the instrumental the time of closure of the abdominal wall; however, no studies have addressed these actions [1,2,4,10,32]. The authors agree on the careful performance of all interventions that could produce an endometriotic nodule in the incision as a preventive measure^1.4^ Gynaecological scar endometriosis is an entity that requires a high level of suspicion to be diagnosed and is important in the differential diagnosis of a mass in a scar in women of childbearing age.

**Figure 7:**
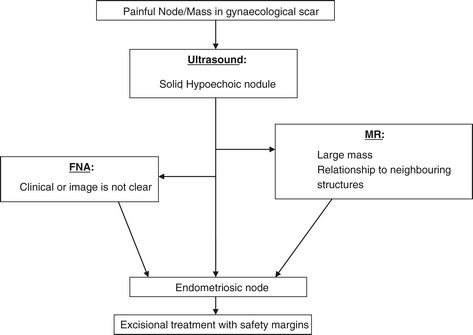
**Algorithm for preoperative diagnosis and treatment.**

We propose an algorithm for preoperative diagnosis and treatment in cases of patients with nodes in gynaecological scars, suggestive of endometriotic origin Figure 7. First, an endometriotic nodule should be suspected to the presence of a painful mass in a previous scar. The classic clinical presentation of gynaecological scar endometriosis is the appearance of a mass in a scar that grows and causes pain with menstruation. Second, imaging techniques such as ultrasound, MR or CT facilitate the diagnostic approach; however, there is no pathognomonic image. Ultrasound is the most cost-effective test; therefore it should be done in the first stage. However, in some cases, CT or MR is required to determine the size and location of a mass as well as its relationship to the adjacent structures. If it is needed; MR will be done in the second stage. FNA is a diagnostic tool in cases in which a node of tumour origin is suspected; however, FNA could lead to complications such as viscus injury and the emergence of a new implant in the puncture site. Accordingly, we propose to do a FNA in the case that the diagnosis by clinical or image is not clear.

## Conclusions

Etiopathogenic hypotheses include the theory of immune tolerance induced by pregnancy; however, most authors support the theory of iatrogenic implantation. The mechanism is most likely explained by a combination of the two hypotheses.

The treatment of endometriosis in gynaecological scars is by resection with safety margins, although in some cases such as those with large masses or concurrent pelvic endometriosis, surgical treatment is combined with hormonal therapy.

No preventive measure has been shown to decrease the risk of endometrial nodules in scars; it appears to be prudent in performing gynaecological interventions that could produce an ectopic endometriotic nodule.

### Consent

Written informed consent was obtained from the patient’s guardian/parent/next of kin for the publication of this report and any accompanying images.
